# Photosynthesis by marine algae produces sound, contributing to the daytime soundscape on coral reefs

**DOI:** 10.1371/journal.pone.0201766

**Published:** 2018-10-03

**Authors:** Simon E. Freeman, Lauren A. Freeman, Giacomo Giorli, Andreas F. Haas

**Affiliations:** 1 Naval Undersea Warfare Center, Newport, RI, United States of America; 2 Underwater Acoustics and Signal Processing Division, U.S. Naval Research Laboratory, Washington D.C., United States of America; 3 Naval Undersea Warfare Center, Newport, RI, United States of America; 4 National Institute of Water and Atmospheric research, Greta Point, Wellington, New Zealand; 5 Department of Marine Microbiology and Biogeochemistry, NIOZ Royal Netherlands Institute for Sea Research and Utrecht University, Texel, Netherlands; University of Auckland, NEW ZEALAND

## Abstract

We have observed that marine macroalgae produce sound during photosynthesis. The resultant soundscapes correlate with benthic macroalgal cover across shallow Hawaiian coral reefs during the day, despite the presence of other biological noise. Likely ubiquitous but previously overlooked, this source of ambient biological noise in the coastal ocean is driven by local supersaturation of oxygen near the surface of macroalgal filaments, and the resultant formation and release of oxygen-containing bubbles into the water column. During release, relaxation of the bubble to a spherical shape creates a monopole sound source that ‘rings’ at the Minnaert frequency. Many such bubbles create a large, distributed sound source over the sea floor. Reef soundscapes contain vast quantities of biological information, making passive acoustic ecosystem evaluation a tantalizing prospect if the sources are known. Our observations introduce the possibility of a general, volumetrically integrative, noninvasive, rapid and remote technique for evaluating algal abundance and rates of primary productivity in littoral aquatic communities. Increased algal cover is one of the strongest indicators for coral reef ecosystem stress. Visually determining variations in algal abundance is a time-consuming and expensive process. This technique could therefore provide a valuable tool for ecosystem management but also for industrial monitoring of primary production, such as in algae-based biofuel synthesis.

## Introduction

Coral reef degradation and algal smothering are caused by human impacts such as overfishing, pollution through nutrient runoff and climate change effects. Algal dominance diminishes the value of reefs [1], is likely permanent [2, 3], and serves as a clear indicator of ecosystem degradation [4]. Timely monitoring of reef state is crucial to quantifying impacts and mitigating ecosystem shifts [5], but present-day assessment methods are resource intensive and infrequent [6]. Because coral reefs are highly soniferous ecosystems where most sound comes from biological processes, ecologists have sought to apply the integrative acoustic monitoring approaches used successfully in terrestrial soundscape ecology [7, 8]. These techniques can yield spatially integrative and long-term continuous observations that can be collected autonomously with inexpensive recorders, but the sources of many reef sounds are challenging to identify [9]. Previously we established positive correlations (Pearsons ρ) between benthic macroalgal cover and aspects of coral reef soundscapes (acoustic pressure spectral density levels in the 2 to 20 kHz band) at 17 sites over a 2570 km transect from Kure Atoll to the Island of Hawaii [10]. These correlations were only observed during the day when overall sound levels were typically lower (Table 1), and correlations of this nature did not exist between sound and any other non-acoustic environmental metric. To further deconvolute the soundscape sources, we performed tank-based passive acoustic experiments with the invasive Hawaiian algae *Salicornia gracilaria*. Ambient sound was recorded in combination with dissolved oxygen and time-lapse photography of algae under daylight and night conditions to investigate the mechanism behind the observed associations of sound with algal presence.

**Table 1:** Correlation between benthic algal cover and coral reef soundscapes. Pearson’s correlation coefficients between the percentage of benthic macroalgal cover at 17 reef sites throughout the Hawaiian Islands and ambient acoustic pressure spectral density in three bands between 2 and 20 kHz, obtained simultaneously at the same locations. Intensity-filtered spectra were averaged over one hour beginning at the indicated times.

Time of day	Acoustic frequency band, kHz	Pearson’s correlation coefficient, ρ
Pre-dawn, 2 h before local sunrise	2–7	0.33
	7–13	0.50
	13–20	0.48
Morning, 1 h after local sunrise	2–7	0.63
	7–13	0.70
	13–20	0.68
Afternoon, 2 h before local sunset	2–7	0.67
	7–13	0.68
	13–20	0.67
Evening, 1 h after local sunset	2–7	0.35
	7–13	0.56
	13–20	0.55

Short-time Fourier analysis on intensity filtered soundscape recordings [10] provide a more spectrally detailed view of individual transient soundscape components (i.e., most reef-based biological sound). Typical Fourier analysis approaches in underwater acoustics integrate over time periods that are much longer than each individual biological sound. While longer integration results in increased frequency resolution, ideal for detecting tonal and/or narrow-band sources, the approach can also spectrally smear multiple transient sounds together and/or reduce peak level estimates through giving equal weighting to quiet periods between transient arrivals. If a persistent environmental noise is present, it can mask some transient biological sounds sampled through this approach. Our approach involved selecting each transient using an intensity filter, ensuring the transform length encompassed only the transient, and then assessing the spectral qualities of each transient individually. As a consequence, reef spectra shown here cannot be directly compared to spectra obtained without intensity filtering. Depending on ambient and preamplifier noise levels, lower-level sounds from individual biological sources may not be discernable at significant distances from the source. However, many of these events occurring over a distributed area create an extended sound source for which propagation models show decreased attenuation with distance [11].

## Results

**Fig 1:**
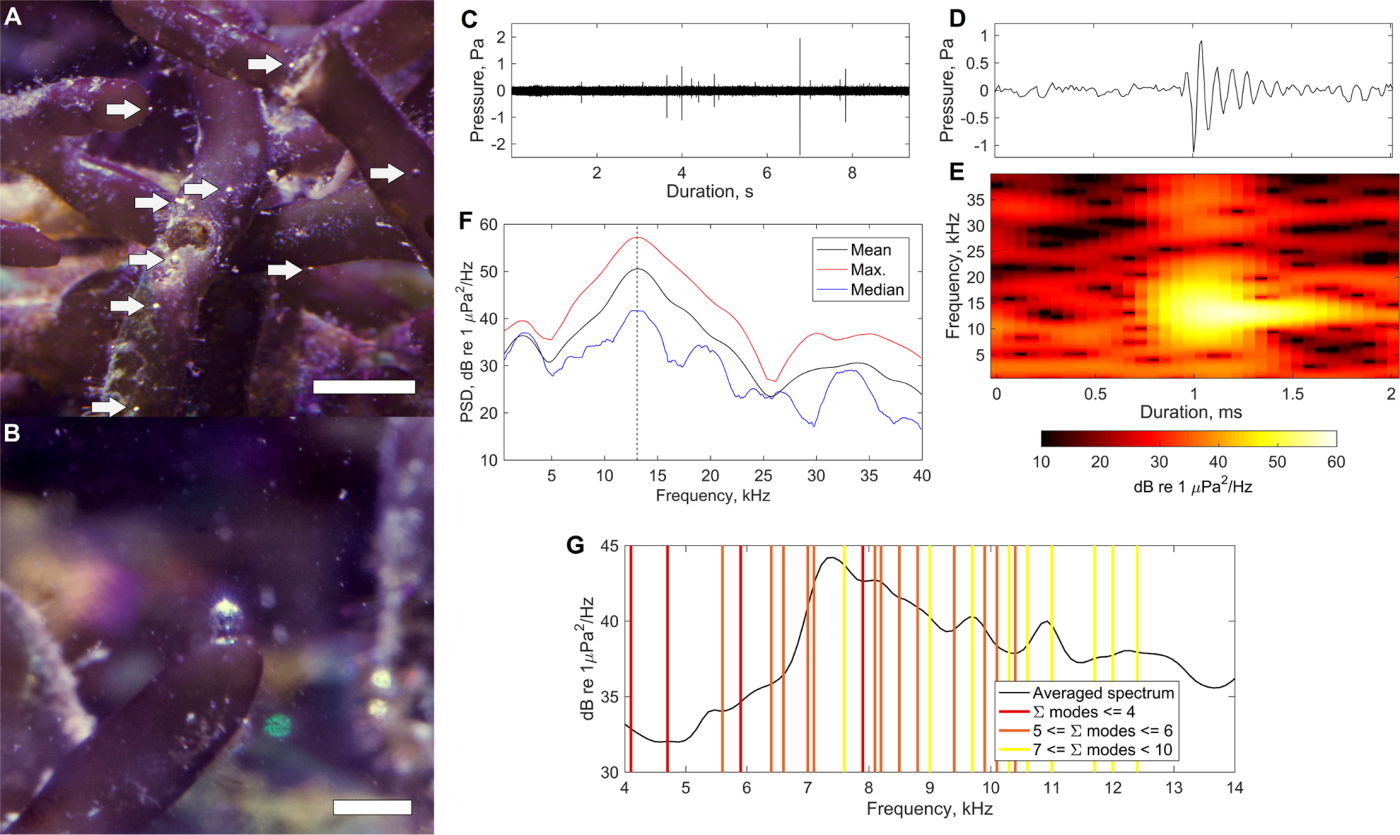
Characterization of bubble sound from algae. (**A**) *Gracilaria salicornia* actively creating gas bubbles during photosynthesis. White arrows indicate the locations of larger bubbles. The scale bar is 5 mm in length (**B**) A closer view of one gas bubble about to detach from the algae surface. The scale bar is 2 mm in length. (**C**) A 9 s time series of ambient sound from inside the aquarium. (**D**) A high-resolution view of a typical transient waveform as shown in (**C**). (**E**) Spectrogram showing the time-varying spectral content of the received waveform in (**D**). (**F**) Pressure spectral density (PSD) estimates showing mean, median and maximum pressure spectral densities of received level from the 2 ms period as shown in (**D**) and (**E**), indicating the spectral peak of the waveform as 13.07 kHz (vertical dashed line). (**G**) An averaged spectrum from transient sounds recorded over one hour overlaid with standing-wave resonant tank modes. Mode sums indicate the sum of mode numbers in the horizontal (length, width) and vertical (depth) directions.

With the onset of photosynthesis during light periods, bubbles could readily be observed with the naked eye on the surface of macroalgae (Fig 1A and 1B). As the bubbles detached from the plant, they created a short ‘ping’ sound. Acoustic recordings of bubble release separated by quiet periods consequently appear as an irregular pulse-train-like time series (Fig 1C). The sound created by perturbation of the bubble during release is naturally transient and decays exponentially (Fig 1D). Oversampled recordings (100 kS·s^-1^) permit more detailed spectral analysis of each sound (Fig 1E) and fundamental frequency estimates for each bubble can be made from the resultant data (Fig 1F). Tank resonance modes [12] were restricted through the presence of algae and the drafted, acoustically absorptive nature of the tank walls and did not appear to substantially influence the recorded spectra (Fig 1G).

**Fig 2:**
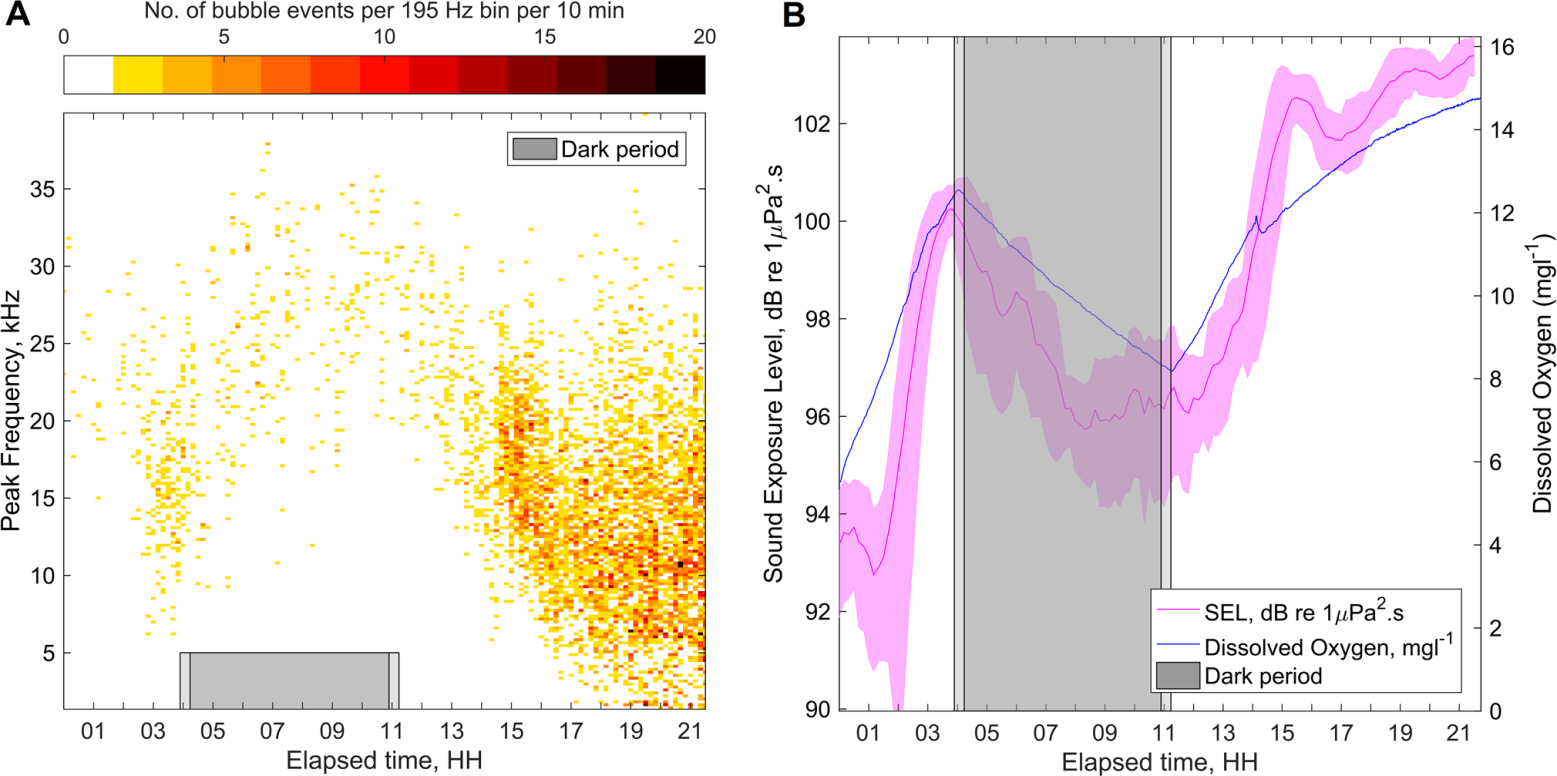
The acoustic response of algae to light over time. (**A**) Ten-minute averaged time-frequency histograms showing the distribution of Minnaert frequencies from bubbles produced by algae with the application and removal of PAR. The color scale indicates the number of bubble events acoustically characterized over a 10-min period, per frequency bin (195 Hz bin width). (**B**) Sound Exposure Level (SEL, 10 min. averages, ± 1 S.E., left axis) and dissolved oxygen concentration (right axis) showing an increase, decrease, and increase of dissolved oxygen and SEL with the application, removal and application of PAR, respectively. The grey regions indicate the period when the light source was removed.

Experiments with *S*. *gracilaria* in controlled laboratory settings demonstrate that sounds produced by algal photosynthesis are similar in nature to components of the soundscape recorded in shallow water regions where *S*. *gracilaria* and other benthic macroalgae species are common. With the intermittent application of Photosynthetically Active Radiation (PAR, *μ* = 381, *σ* = 67 μmol·m^-2^s^-1^, roughly equivalent to the available level at water depths of 10 m on a cloudy day in the equatorial pacific) the link between photosynthesis, dissolved oxygen and acoustic emissions through bubble formation becomes apparent (Fig 2 and S1 Fig). Over the duration of PAR application both the number of bubbles (R^2^ = 0.76, S2A Fig) and their mean size (R^2^ = 0.47, S2B Fig) increased with dissolved oxygen levels. The process was reversed with the removal of light. These increases in the size and rate of bubble formation during the illuminated period lower the frequency distribution of sounds produced by the algae (Fig 2A) and cause an increase in the Sound Exposure Level (SEL, R^2^ = 0.82, Fig 2B and S2C Fig), a measure analogous to acoustic work. The onset of bubbling rate and size increase from the application of light was delayed due to the time required for photosynthesizing algae to build supersaturation conditions in the surrounding water, and for bubbles to reach a size large enough to create sufficient buoyancy force to detach from the algae. After removal of the light source there was a comparable delay in the reduction of bubble production and sound, due to bubbles remaining on the algae and saturation state remaining above 100 percent.

**Fig 3:**
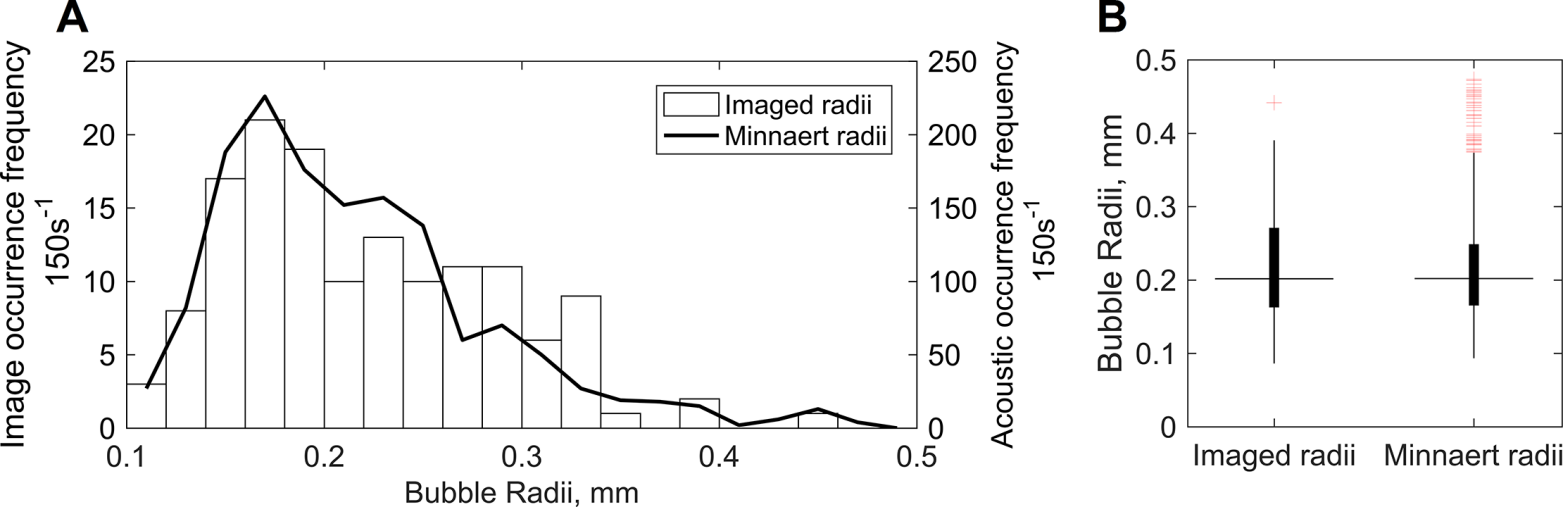
Size distribution comparisons between bubble radii distributions simultaneously derived through photography and passive acoustic recording. (**A**) Bubble size distributions obtained from photographically imaged measurements (histogram, left axis) and Minnaert radii (solid line, right axis) every 150 s over a one-hour period of active bubble formation. (**B**) Boxplots indicating 25^th^, 50^th^, 75^th^ percentiles and 5^th^ and 95^th^ percentile outliers (crosses) of optically imaged and acoustically derived bubble radii distributions over the same period.

A comparison between acoustically derived bubble size distributions and photographically obtained measurements of bubble size (Fig 3) optically validates the acoustic estimation of algae-driven bubble radii (99 percent significance: no evidence the distributions were unequal, Wilcoxon 2-sided test, p = 0.9087).

## Discussion

Algae release oxygen as a byproduct of photosynthesis. While these waste molecules are formed intracellularly in solution, the nucleation of oxygen gas bubbles on the surface of macroalgal tissue takes place when localized supersaturation of dissolved oxygen occurs at a nucleation site. Depending on the timescale of bubble formation and the total gas tension, the diffusion of nitrogen will also contribute to total bubble volume. Previous work has shown the relationship between oxygen bubble production by algae and oxygen supersaturation at their surface microenvironment [13]. The bubbles grow with the addition of more waste oxygen produced by photosynthesis and nitrogen diffusion, ultimately separating from the algae through a combination of buoyancy and surface tension forces. As a spherical bubble is perturbed through release, it oscillates in volume and pressure with exponential decay at a frequency inversely proportional to its radius *R*_0_, the roots of the specific heat ratio of the gas *γ* and ambient fluid pressure *P*_*fl*_, and the inverse root of ambient fluid density, *ρ*_*fl*_, a relationship first derived by Minnaert [14] and now referred to as the Minnaert frequency [15] *ω*_*Minn*_:

$$\omega_{Minn}=\frac{1}{R_{0}}\sqrt{\frac{3\gamma P_{fl}}{\rho_{fl}}}$$ (1)

This approximation was first developed with the assumption of negligible heat flow (adiabatic conditions) and negligible surface tension, but has since been shown to be a good approximation for bubbles of radii between 30 nm to 300 μm. Consequently, passive acoustic estimates of bubble volume can be made if the water properties and depth at which bubble separation occurs are known.

Our observations demonstrate the mechanism behind previously established correlations between components of underwater soundscapes and the relative abundance of macroalgae on Hawaiian coral reefs [10]. Although ecologically distinct, bubble production is ubiquitous in sea grass beds [16], and can also be observed in marine algal species [17]. Thus, the bubble production mechanism is not specific to *S*. *gracilaria* and may be used as a general indicator of photosynthetic activity. Ultrasonic emissions from terrestrial plants are also driven by bubble processes, but the underlying mechanism is water starvation rather than photosynthesis [18].

The contribution of algal sound to soundscapes may be isolated from other ubiquitous sound sources though matched filtering, directional receivers and similar techniques. The observed size distribution and thus corresponding spectral distribution of bubble sound (Fig 3A), and the a-periodic and un-clustered nature of acoustic emission time series (Fig 1), are not shared by any other bubble related process in shallow-water environments, including breaking waves [19]. While active acoustic techniques have been used to estimate marine plant biomass [16, 20], our finding introduces the possibility of noninvasive sensing methods that can quantify relative primary production rates in addition to presence/absence, although further work in disentangling confounding factors is necessary. The relationship between bubble formation processes and gas transfer mechanisms has been well studied [13, 21–22] providing a background for the application of bubble science to underwater ecological sensing.

The findings presented here show that algae are capable of producing sound under normal circumstances and that sound may explain a correlative association previously discovered between sound in the 2 to 20 kHz band and algal cover on coral reefs [10]. However, a great deal of further work is required before a passive acoustic tool could be developed for quantifying bubble output from photosynthesis, in-situ primary productivity and algal abundance. A number of caveats in our analysis must be considered and results from soundscape correlation and tank experiments should be considered limited in scope until these caveats are resolved. Underwater soundscapes, especially in the vicinity of a shallow coral reef, are extremely complicated acoustic environments containing sound sources of many types [23]. A number of spatio-temporally variable physical mechanisms that influence the production and propagation of sound from source to receiver also exist.

**Fig 4:**
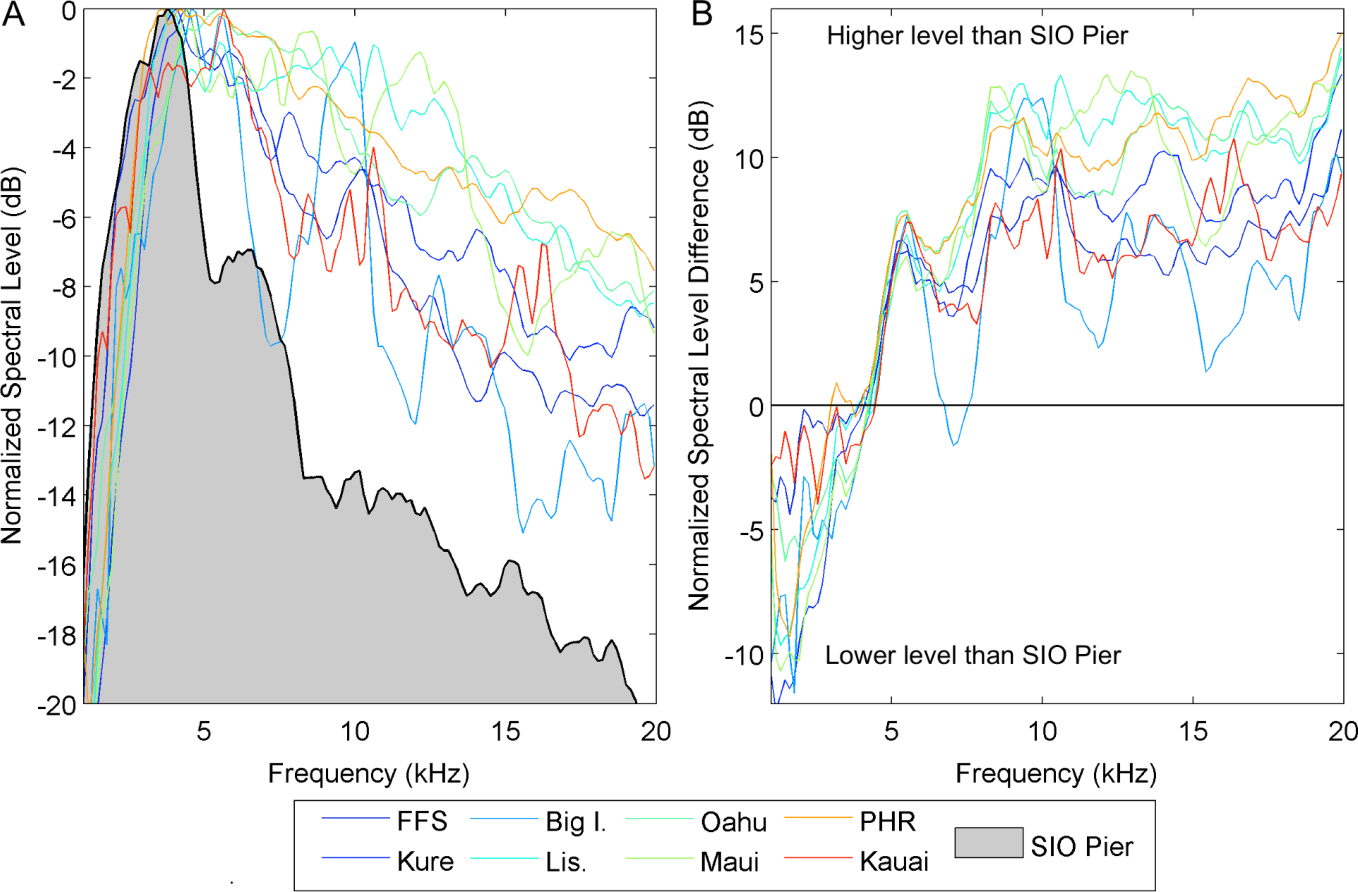
Spectral comparisons between Hawaiian reef soundscapes and the snapping-shrimp dominated soundscape at the Scripps Pier in La Jolla, CA. (**A**) Normalized, intensity filtered pressure spectral density levels recorded at midday local time during sunny days on shallow Hawaiian reefs (colored lines) plotted with similarly filtered levels obtained during crepuscular chorusing of snapping shrimp communities on the Scripps pier (black line with grey shading underneath). (**B**) Logarithmic spectral level differences between crepuscular pier and midday Hawaiian reef soundscapes in Fig 4A. Note that 3 dB represents a doubling or halving of the spectral level difference in each frequency bin. Hawaii locations are identified as follows: FFS–French Frigate Shoals; Kure–Kure Atoll; Big I.–Ke’ei Beach, Big Island Hawaii; Lis.–Lisianski Island; Oahu–Lai’e Beach, Oahu, Hawaii; Maui–La Perouse Bay, Maui, Hawaii; PHR–Pearl and Hermes Reef; Kauai–Tunnels Beach, Kauai, Hawaii.

The most notable interference may be snapping shrimp noise. While the sound generation mechanism of these shrimp involves cavitation [24] and is thus spectrally unique from the relatively narrow-band bubble emissions discussed here, source levels and rate of occurrence can be so high [25–27] that they are capable of ‘drowning out’ other sound sources for protracted periods of time. Furthermore, the spectral, temporal frequency, and diurnal behavior of snapping shrimp vary depending on the location and time of year [28–29, 26–27]. While the frequencies produced by photosynthetic bubbles are within the band of sound produced by snapping shrimp, spectral analysis of sound from shrimp-dominated and coral reef soundscapes reveal spectral differences that suggest other biological contributors can be spectrally evaluated even in the presence of shrimp noise. Bubbles produce transient sounds that contain energy in a relatively narrow frequency band. Due to the higher likelihood of smaller bubbles, it is less likely that bubbles would add significant energy at lower frequencies (i.e., below 5 kHz). Conversely, sound produced by snapping shrimp is broad-band but weighted toward a peak between 4 to 6 kHz [25]. The integration of snapping shrimp sounds over time can produce a low-frequency-weighted spectrum with monotonic decay. Conversely, some reef soundscapes may create higher-frequency-weighted spectra that may include distinct spectral peaks (Fig 4A). Limited comparisons between a snapping-shrimp-dominated soundscape and those correlated with algal dominance in Table 1 show a variation in band levels between 5 and 20 kHz between sites. In this case, the shrimp-dominated soundscape is the Scripps Pier in La Jolla, Ca. Periodic cleaning of encrusting sponges on the pilings reveals large communities of snapping shrimp. Care should be taken when evaluating this comparison as the behavior and sound of snapping shrimp may vary between Hawaii and San Diego. However, the Scripps Pier is relatively unique in that no reefs exist within half a mile of the pier. Recordings were made on a calm day with little swell. The comparison shows that in daytime reef soundscape levels were between 5 to 12 dB higher above approximately 5 kHz. Barring differences in frequency-dependent attenuation or shrimp sound characteristics, the comparison suggests an additional source of higher frequency sound present at the reef sites (Fig 4B). The high source levels emitted by snapping shrimp drive the prevailing thought that they are the overwhelmingly dominant bioacoustic source in coastal underwater ecosystems. However, our suggestion that individually quieter biological sources of sound are detectable in the presence of snapping shrimp is not unprecedented. Chorusing from sea urchin grazing is known to contribute to soundscape spectra off the coast of New Zealand [30] and hermit crabs have been spectrally matched to soundscapes from environments in which both they and snapping shrimp are plentiful [9]. Nevertheless, before accurate quantification of algal photosynthetic activity can be attempted in the field, the behavior and acoustic characteristics of snapping shrimp in the area must be understood to create validated acoustic signal processing algorithms capable of differentiating between shrimp noise and algal sound.

**Fig 5:**
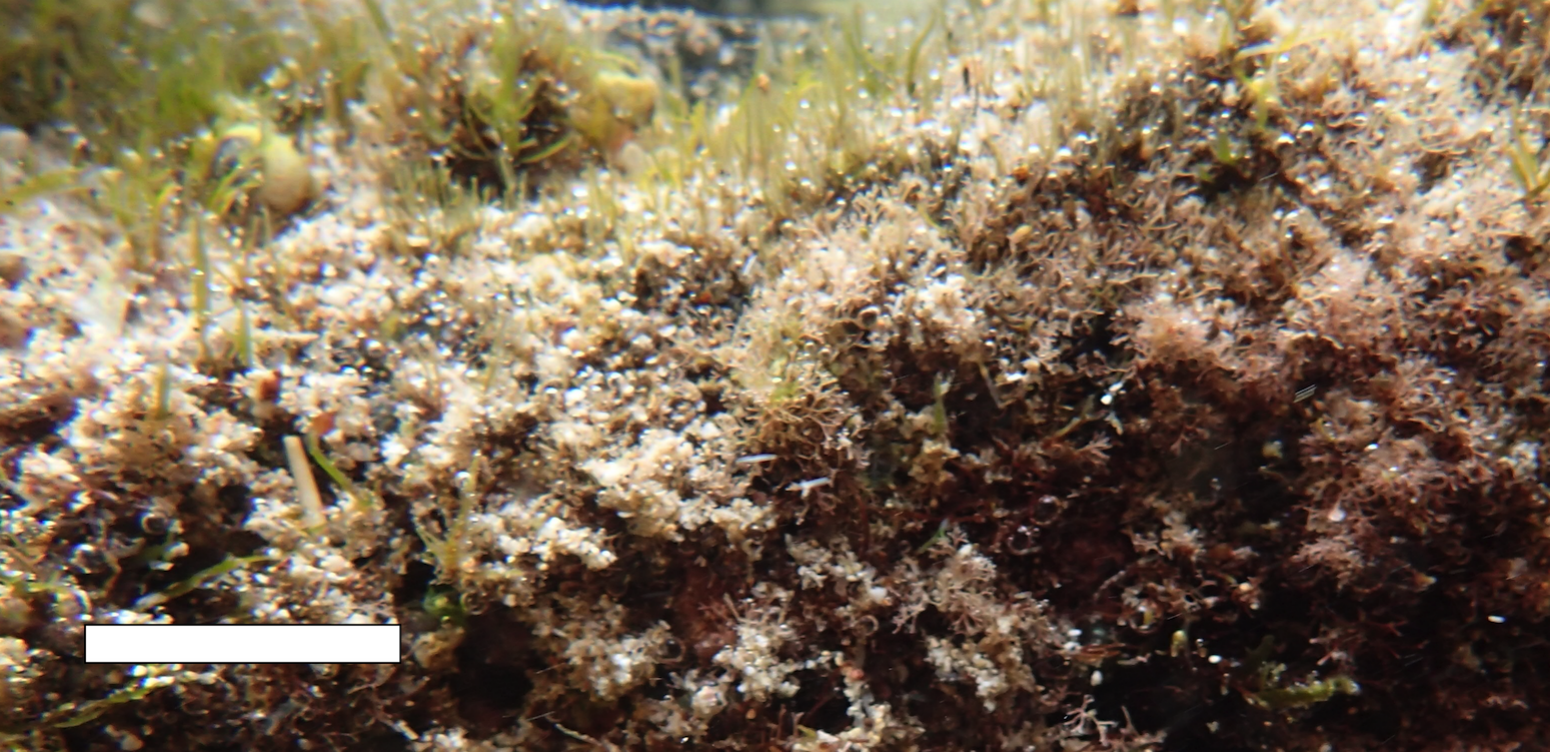
Bubbles from photosynthesis present on shallow algal turf subject to swell. Note the movement of suspended particles during the camera exposure period of 1/125 sec. Scale bar indicates 2 cm. Image courtesy Florybeth La Valle, University of Hawaii.

Another confounding factor may be the influence of water movement on bubble formation and retention. Bubbles will not form if water is continually swept from the algae and oxygen saturation state never exceeds 100 percent. Secondly, if bubbles are formed, they may be prematurely removed through wave action, decreasing the size distribution of the bubbles and shifting the frequency distribution higher. The mechanisms governing benthic oxygen saturation state and the effects of water movement on bubble retention are poorly understood. Benthic algae reside at least partially within the fluid boundary layer, especially in rugose environments, and may be somewhat protected from flow. The structure of algal filaments may also assist or retard the removal of bubbles by wave action, meaning that aspects of sound production are likely species-specific. While bubble retainment by algae in current remains to be quantitatively investigated, Fig 5 shows typical turf algae in a shallow reef environment off Hawaii’s Big Island on a sunny day. Fig 5 demonstrates that in very shallow water subject to wave action, bubbles from photosynthesis may be retained if sufficient algal structure exists. Further acoustic, flow, and bubble physics analysis, along with an investigation of biological interaction (both floral and faunal) are required to quantify and understand the impact water movement has on bubble formation and the consequent acoustic emissions by algae in these scenarios.

The probability that too many transient sounds arrive at the receiver during one FFT integration period for spectral analysis on individual sounds to be performed increases with the size of the reef, the nature of propagation pathways from sources to the receiver, and the acoustic productivity of the benthic community. As the range between any given source and the receiver increases, the probability that multipath arrivals are interpreted as separate signals also rises. However, the spectral content of multipath arrivals is less likely to differ significantly from the direct arrival when compared to other sound sources. Future data collection with a spatial filter such as a directional receiver or hydrophone array can limit the area over which passive acoustic surveys are performed, simplifying the challenges associated with multipath and the large number of sources.

Sound is the most efficient radiation underwater and we continue to discover that important biological components of many aquatic ecosystems are both sensitive to [31] and produce acoustic emissions. Contributions to biological soundscapes on coral reefs include sound deliberately produced by fishes and invertebrates for communication [32] and defense [25], or inadvertently through feeding [30] and movement [9]. To date these sounds have been used to study the behavior and distribution of marine mammals and fish [32–33], to understand ambient noise levels in the ocean [34–35], and to assess the impacts of anthropogenic noise on marine environments [36]. Soundscape measurements are also effective at night when benthic activity is greatest [9] and passive optical techniques are difficult or impossible to implement [37]. In concert with these acoustic observations, a volumetrically integrative, remote and noninvasive method of measuring primary productivity outputs may lead to more accurate, persistent and less expensive surveys of ecological state in shallow water, the majority of which are currently performed *in-situ* through diver-based optical methods. Quantifying primary productivity through passive acoustic monitoring could become a technique that may not be limited to coral reef environments. Macroalgae are the dominant benthic primary producer in many temperate coastal and freshwater environments that are more difficult to survey optically due to reduced water clarity. Passive acoustic monitoring of ebullition has been utilized in the monitoring of chemical reactions [38]. A semi-real-time and volumetrically integrative approach can be adopted in biofuel-generating algal reactors using similar methods. The sensing technique proposed here is restricted neither to in-situ observations nor to marine environments but may apply basically to all aquatic environments in which macroalgae grow.

## Materials and methods

### Background data

The summary data shown in Table 1 were collected in the Northwest Hawaiian Islands (NWHI) during a NOAA Remote Areas Monitoring Program Cruise HA-12-04 on the R/V Hi’Ialakai during August 2012 and from sites accessible from shore on the Main Hawaiian Islands during September-October 2012. Permits were obtained for conducting research in the Papahānaumokuākea Marine National Monument (PMNM-2012-029) and the Main Hawaiian Islands (Department of Land and Natural Resources special activity permit 2012–83). Acoustic data were collected using a single hydrophone Loggerhead Instruments DSG Ocean recorder configured with a sampling frequency of 80 kSs^-1^ and an electronic gain of 20 dB. Simultaneous with acoustic recorder deployments, benthic phototransects were taken in accordance with NOAA Coral Reef Ecosystem Division rapid ecological assessment protocol [39]. Pearson’s correlation coefficient, ρ, between all phototransect-derived environmental variables, and band-limited pressure spectral density estimates, were calculated over data from all field sites for which acoustic and phototransect data were available. Metrics for which ρ values were greater than 0.6 with p < 0.001 (Bonferroni correction) were considered to be sufficiently correlated. Correlations of particular interest were those that appeared to form a distinct pattern across several metrics (i.e., similarly high correlation across adjacent acoustic bands, combined with a consistent temporal pattern). A full description of the data collection and processing methods associated with Table 1 is available in the associated publication [10].

### Summary of the experiment

Tank-based experiments were carried out at the Hawaii Institute of Marine Biology at Coconut Island, Kaneohe Bay, Hawaii. Ten kilograms (wet) of the invasive red algae *Gracilaria salicornia* were collected by the Department of Aquatic Resources (DAR) of the State of Hawaii in Kaneohe Bay and stored in a 1 m diameter seawater holding tank with a steady filtered (100 um) ocean water flow. One kilogram of algae was visually inspected to remove any associated fauna and relocated to a smaller, plastic, opaque-sided rectangular tank with internal dimensions of 550 x 300 x 300 mm. Seawater was used to fill the tank to a depth of 250 mm and a 50 mm layer of algae was positioned on the floor of the tank between 200 and 250 mm depth. The algae remained negatively buoyant throughout the experiment. An aquarium light (Radion XR30w G4 pro) was positioned centrally over the tank and 228 mm (9 in.) above the water surface. All testing was conducted in a dark room with the aquarium light being the only source of photosynthetically available radiation (PAR). The color temperature was set at close to that of natural sunlight on a clear day (5500 K), at which the light operated at 55 percent of maximum output. Software limitations required a warm-up and cool-down period of 30 min before and after full-strength application, during which light levels were linearly ramped up and down between zero and 55 percent. Mean PAR level was obtained from measurements at fifteen equally spaced locations at algae equivalent depth using a calibrated LI-COR Li-193 spherical underwater quantum sensor and a LI-COR Li-250 light meter. Note that the mean PAR level obtained (381 μmol·m^-2^s^-1^) is far below what is expected near the surface on a calm sunny day in the tropics [40]. The experiment was started with algae being brought out of a 24-hour period of darkness and exposed to PAR as described above. After 3 h and 45 min, light levels were reduced to zero. After 7 h of darkness, light levels were increased back to the levels described above until the conclusion of the experiment after 10.5 h of light. The algae were then removed from the tank and disposed of in accordance with DAR procedure.

### Acoustic recordings

Acoustic data were collected with a High Technology Inc. HTI-92-WB hydrophone, equipped with a low-power, high sensitivity preamplifier. Hydrophone and preamp system sensitivity was -144.8 dB re 1 VμPa^-1^ over the 2 Hz to 40 kHz band. The hydrophone was positioned to one side of the tank at 125 mm depth, 60 mm from the nearest tank wall. The preamplifier was powered by a variable DC power supply set at 10 V and drawing 2 mA. Data were acquired using a National Instruments® USB-6366 data acquisition module connected to a laptop computer running National Instruments® LabView® 2013 (SP1) and customized script. Time-stamped data were sampled at 100 KSs^-1^ at 16-bit depth in discrete 10 s intervals, pausing for a short time in between to save the data and verify that the sampling frequency was accurate. The recordings were processed in Mathworks Matlab® software. Before analysis, a high-pass, 5^th^ order Butterworth filter (stop band: 0–0.5 kHz, pass band: 0.8–50 kHz was applied to remove DC offset, 60 Hz electrical noise or low frequency noise from passing vehicles and other sources.

### Acoustic data processing

The peaks in amplitude associated with bubble separation ‘pings’ were identified from pre-filtered acoustic data if absolute peak amplitudes were greater than 5 σ from preamp self-noise levels. A 10 ms window around each of these peaks was isolated for further analysis. Information regarding the number of qualifying peaks and the temporal distribution of inter-peak periods within each 10 s file was retained. In order to calculate the sound exposure level (SEL) of bubble sound, the time duration of each air bubble “ping” was determined as the time between the 5^th^ and 95^th^ percentiles of the cumulative energy within the 10 ms window. SEL values were averaged over successive 10-min periods. Spectral analysis was performed on the time series between the 5^th^ and 95^th^ percentiles using a Kaiser-Bessel-windowed (β = 2.5π) 512-point FFT, overlapped 90 percent, meaning a bin width of 195.3 Hz and an overlapped spectral estimate made every 512 μs. The frequency bin containing the highest pressure spectral density level above 1 kHz, temporally matched to the amplitude peak in the corresponding time series, was determined to best represent the dominant frequency of each bubble separation ping. Regression of log-linear relationships and calculation of residuals was conducted using a simple logarithmic (base ten) model for the relationship between SEL and dissolved oxygen, and a natural logarithmic model for the relationship between the number and mean size of bubbles and dissolved oxygen. Residuals were calculated using linearized values.

### Determination of bubble size

Estimates of bubble radii (R_0_) corresponding to each peak frequency were made using the Minnaert equation (1). For *R*_0_ to be a good approximation for the true radii of a freely oscillating bubble, the bubble radius must satisfy the following conditions: that the bubbles are “acoustically small”, the geometric mean of bubble radius and acoustic wavelength is large compared to the viscous boundary layer, the bubbles are “thermally large”, and the Laplace pressure is much less than the equilibrium pressure in the liquid (20).

### Dissolved oxygen, pH conductivity, and temperature data

Dissolved oxygen (DO), pH, conductivity, and temperature were measured in 60 s intervals over the duration of the experiment. All non-acoustic sensors were part of a Manta+ 20 multi-probe system placed inside the experiment tank opposite the hydrophone. DO was assessed with an optical dissolved oxygen sensor provided by the Hamilton company (HDO) with a resolution of 0.01 mgL^-1^ or 0.1% saturation. pH was assessed via an electrolyte filled glass sensor next to a reference electrode. For conductivity, a four electrodes sensor was used while the temperature was assessed with a thermistor. Additional specifics are provided by the manufacturer [41]. While essential for the experiment, the oxygen sensor produced two types of acoustic noise that were additionally filtered from the acoustic data before analysis. The first type of noise was a startup transient that occurred upon system power-up every 60 s. A matched filter was used to exclude 0.2 s portions of recordings in which the transient was present. Secondly, a steady-state doubling of high frequency noise levels was emitted by the sensor for approximately 8 s after the startup transient. These recording periods were identified using a Hilbert-transform-based threshold detection algorithm and excluded from analysis.

### Optical validation

Bubble radii were directly measured from high resolution photographs taken using a Canon SL1 single lens reflex camera equipped with a Canon 100 mm F2.8 L macro lens (160 mm effective) mounted on a tripod outside of the aquarium and aimed inward through a transparent window. A scale rule bar was inserted into the aquarium and placed across the field of view, orthogonal to the lens axis and within the depth of field. Only bubbles that were in focus within the depth of field of each image were analyzed. Photographs were taken every 150 s for one hour during a period when algae were observed to be actively producing bubbles. Radii measurements were obtained in post-processing using ENVI software, measured by pixel width and converted to μm using the scale bar in individual photographs.

## Supporting information

S1 Fig Chemical and acoustic responses of algae to changes in photosynthetically available radiation (PAR). All subfigures are time-aligned. (**A**) Dissolved oxygen time series. (**B**) Acoustically determined bubble counts per minute. (**C**) Acoustically determined mean bubble radius per minute.
(TIFF)

S2 FigLog-linear regression plots of acoustic emissions from photosynthesizing algae.(**A**) No. of bubbles per minute against dissolved oxygen levels (R^2^ = 0.76, exponential coefficients *α* = -2.54, *β* = 0.43). (**B**) Mean bubble radii per minute against dissolved oxygen levels (R^2^ = 0.47, exponential coefficients *α* = -3.85, *β* = 0.19). (**C**) 10-minute Sound Exposure Level against dissolved oxygen levels (R^2^ = 0.82, exponential coefficients *α* = 83.15, *β* = 1.39). The coefficients may be applied to an exponential regression of linear parameters *x* and *y* as follows:
$y=e^{\alpha }∙e^{\beta x}$(2)(TIFF)
