# Supplementary figures and images for: Photosynthesis by marine algae produces sound, contributing to the daytime soundscape on coral reefs
Source: PLoS One. 2018 Oct 3;13(10):e0201766. doi: 10.1371/journal.pone.0201766 (PMC6169855; doi:10.1371/journal.pone.0201766)

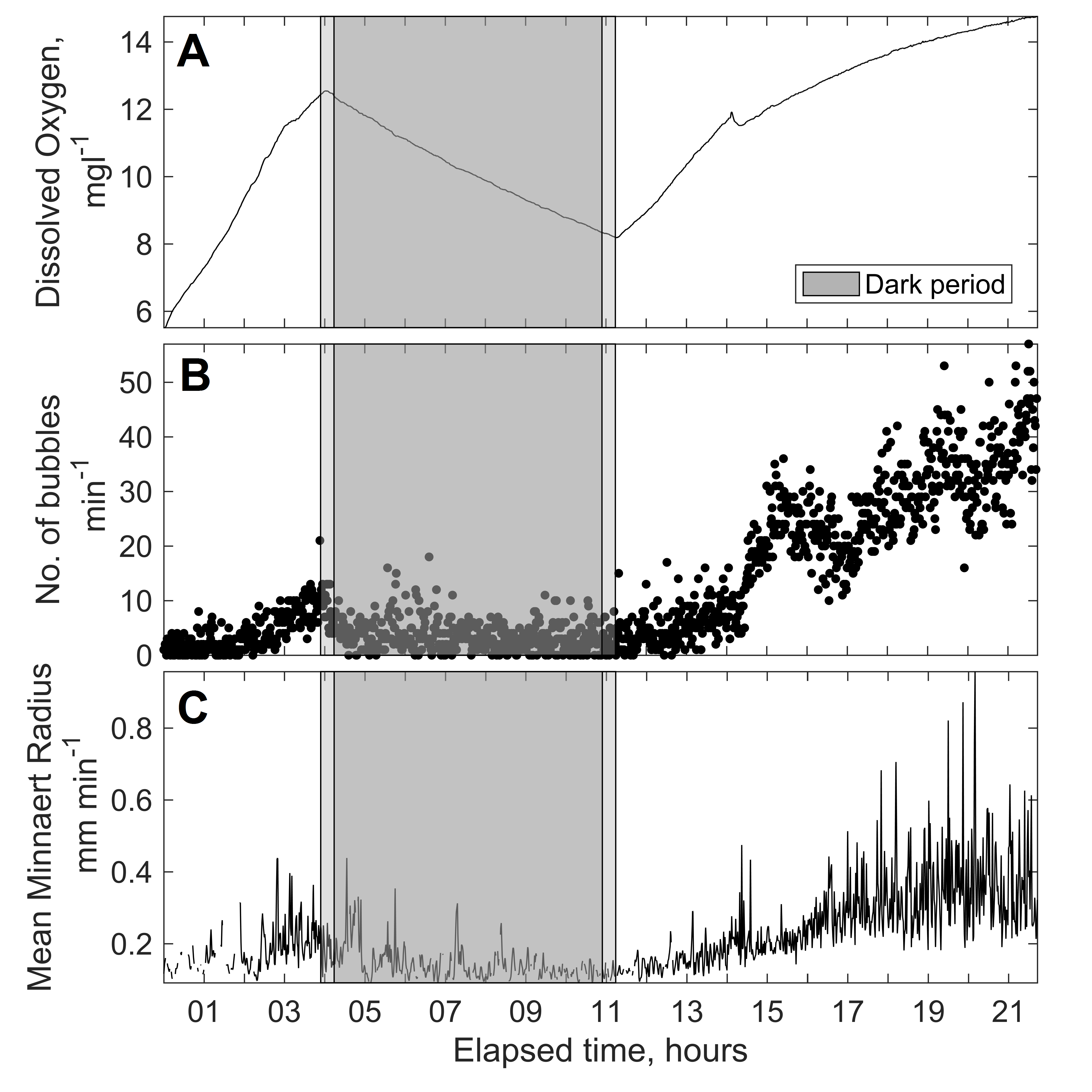

Supplement: S1 Fig — All subfigures are time-aligned. (A) Dissolved oxygen time series. (B) Acoustically determined bubble counts per minute. (C) Acoustically determined mean bubble radius per minute. (TIFF) [file pone.0201766.s001.tiff]

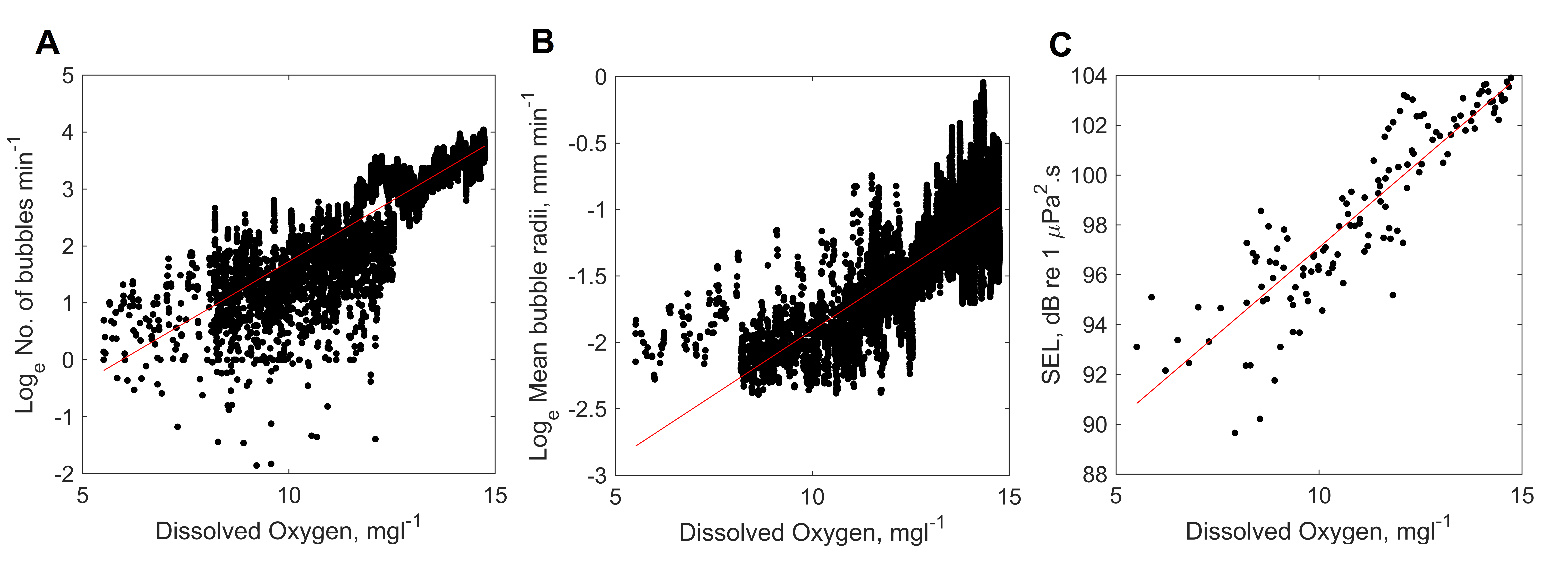

Supplement: S2 Fig — (A) No. of bubbles per minute against dissolved oxygen levels (R2 = 0.76, exponential coefficients α = -2.54, β = 0.43). (B) Mean bubble radii per minute against dissolved oxygen levels (R2 = 0.47, exponential coefficients α = -3.85, β = 0.19). (C) 10-minute Sound Exposure Level against dissolved oxygen levels (R2 = 0.82, exponential coefficients α = 83.15, β = 1.39). The coefficients may be applied to an exponential regression of linear parameters x and y as follows: y=eα∙eβx(2) (TIFF) [file pone.0201766.s002.tiff]
